# Young Human-Derived Microbiota Ameliorates Cognitive Decline and Reproductive Senescence in Aged Mice

**DOI:** 10.3390/nu18081193

**Published:** 2026-04-10

**Authors:** Xiaoying Zhang, Fang Chen, Yinghua Luo, Daotong Li, Junfu Ji, Lingjun Ma, Chen Ma, Xiaosong Hu

**Affiliations:** College of Food Science and Nutritional Engineering, National Engineering Research Center for Fruit and Vegetable Processing, Key Laboratory of Fruit and Vegetables Processing Ministry of Agriculture, Engineering Research Centre for Engineering Vegetables Processing, Ministry of Education, China Agricultural University, Beijing 100083, China; xiaoying_zhang2021@163.com (X.Z.); chenfangch@sina.com (F.C.); luoyinghua@cau.edu.cn (Y.L.); lidaotong@cau.edu.cn (D.L.); junfu.ji@cau.edu.cn (J.J.); lingjun.ma@cau.edu.cn (L.M.)

**Keywords:** aging, fecal microbiota transplantation, cognition, reproduction, metabolomics

## Abstract

**Background/Objectives**: Age-related gut microbiota dysbiosis leads to systemic oxidative stress, chronic inflammation, and multi-organ functional decline. However, there is limited evidence supporting microbiota-based therapies for aging. This study aimed to examine the effect of gut microbiota from young donors, particularly those with increasing *Bifidobacteria* levels through dietary intervention, on age-related declines in fertility, cognition, and reproduction. **Methods**: We conducted experiments using gut microbiota from young human donors, with or without pre-conditioning with barley leaves (BL), to transplant into aged male mice. Hippocampal metabolome and behavioral assessments were used to identify differences in recognitive regulation during aging. Moreover, testis tissue, semen quality, and offspring studies were determined to investigate the beneficial effects on fertility and underlying mechanism. **Conclusions**: This preliminary dietary treatment promotes the growth of *Bifidobacterium* in aged recipient mice. Aged male mice received young fecal microbiota transplants (yFMTs), BL-conditioned yFMTs (BLyFMTs), and a combined treatment of BLyFMT plus recipient BL supplementation. The combined approach significantly increased intestinal *Bifidobacterium* levels and effectively restored hippocampal metabolomic profiles and cognitive behavior. Additionally, yFMT-based treatments mitigated structural damage to the seminiferous tubules and prevented the germ cell depletion. Consistently, those interventions improved sperm quality and mechanistically enhanced hypothalamic–pituitary–gonadal (HPG) axis activity in aged recipients. These findings highlight *Bifidobacterium* as a key factor in microbiome-driven rejuvenation, enhancing the effectiveness of yFMTs in addressing aging-related declines.

## 1. Introduction

Aging is a complex biological process that affects not only our physical functions but also the composition and function of gut microbes over time [1,2]. During aging, the diversity of the gut microbiota typically declines, which is reflected in a decrease in the production of beneficial gut-derived metabolites [3]. This imbalance occurs in the gut microbiota, which leads to chronic inflammation, oxidative stress, cognitive decline, and reproductive dysfunction [4]. These age-related changes may alter the microbial metabolic profile, increase the concentration of gut-derived metabolites in the bloodstream, and directly or indirectly affect the metabolism of various organs in the host [5]. Fecal microbiota transplantation (FMT) is a novel, microbiota-based therapeutic approach that has been shown in clinical and research studies to reverse an imbalanced gut microbiota and the age-related differences [6,7]. Furthermore, animal studies have demonstrated that FMT from young donors to aged recipients has resulted in improved glucose metabolism, reduced systemic inflammation, and enhanced intestinal barrier function [8,9]. Even more intriguingly, this rejuvenating microbiota transplantation has demonstrated protective effects against behavioral deficits and reproductive dysfunction by modulating the gut–brain and gut–gonadal axes, as well as the hypothalamic–pituitary–gonadal (HPG) axis [10,11,12]. However, the clinical application of this approach remains constrained by multiple challenges, including an incomplete understanding of the underlying mechanisms, high treatment costs, uncertainties of optimal timing and routes, limitations of beneficial effects, as well as concerns over long-term efficacy, tolerability, and safety [13,14]. Moreover, the efficacy of conventional FMT remains suboptimal in elderly recipients, likely due to incomplete engraftment of beneficial bacteria and an ongoing inflammatory response in the host that resists microbial remodelling [15]. Age-associated increases in intestinal permeability and systemic inflammaging may create a hostile microenvironment that limits the colonization resilience of transplanted commensals [16]. *Bifidobacterium*, a health-promoting bacterial genus and core constituent of the human gut microbiota, exhibits a dynamic age-related decline. Typically, increasing age is associated with gut barrier dysfunction and immune senescence, accompanied by a significant reduction in *Bifidobacterium* abundance. Especially the *Bifidobacterium longum* (*B. longum*), which is a representative species of relevance for investigating host–microbe coevolution within the intestinal ecosystem [17,18]. It exhibits an age-associated decline, particularly pronounced in individuals with pronounced physiological impairment. Increasing evidence suggests that health outcomes associated with specific microbial communities can be attributed to particular microbial strains [19]. However, the long-term stability of *Bifidobacterium* engraftment is critically affected by the frequency of FMT, lifestyle, dietary habits, and the immune state of recipients [20]. Therefore, combining targeted dietary interventions might enhance FMT outcomes. Dietary fibers serve as a carbon source that regulates intestinal bacteria and influences human health, particularly the growth of *Bifidobacterium* [21]. We hypothesize that the combined strategy could potentially strengthen the resilience of the youthful microbiota after transfer and amplify its protective effects on the decline of age-sensitive systems. In this context, our study investigates whether supplementing young donor FMT with augmented *Bifidobacterium* can better preserve *Bifidobacterium* vitality and subsequently improve neurocognitive and reproductive outcomes in naturally aged male mice. To this end, the present study established five experimental groups: Young, Aged, yFMT, BLyFMT, and BLyFMT + BL. We systematically evaluated the effects of these interventions on cognitive behavior, hippocampal metabolomic profiles, testicular histology, sperm quality, and reproductive competence. Furthermore, we explored the underlying mechanisms involving oxidative stress, inflammation, and HPG axis activity. Our study provides a comprehensive assessment of the synergistic potential of BL-conditioned yFMT in combating multi-system aging.

## 2. Materials and Methods

### 2.1. Study Design

The barley leaf (*Hordeum vulgare* L.) powder was prepared from young leaves harvested at the 20–30 cm growth stage and processed using ultra-low-temperature airflow wall-breaking technology (300-mesh). The nutritional composition of barley leaf powder used in our study is listed in Supplementary Table S1. Barley leaf, rich in dietary fiber, chlorophyll derivatives, and polyphenols, exhibits potent antioxidant, anti-inflammatory, and prebiotic properties. Moreover, its prebiotic components, such as β-glucans and Insoluble fiber, also exert promising therapeutic and functional potential [22,23,24,25,26,27]. Recent studies suggest its capacity to enrich *Bifidobacterium* through fermentation in an in vitro human colonic microbiota model [28]. Here, we hypothesize that barley leaf intervention, applied both to young human donors (to shape a protective microbiota) and to aged mouse recipients (to reinforce a favorable metabolic niche), will synergistically amplify the efficacy of yFMT in rescuing age-associated deficits. The stool samples were collected from healthy young volunteers either without (control) or following a 60-day BL dietary intervention.

A total of 40 male C57BL/6 mice (2 months, *n* = 8) and aged C57BL/6 mice (14–15 months, *n* = 32) used in this study were purchased from Beijing Vital River Laboratory Animal Technology Co., Ltd. (Beijing, China).

Animals were habituated to the animal facility and were kept under a 12 h light–dark cycle at 21 ± 1 °C and humidity of 55 ± 10%. Food and water were given ad libitum. The mice were randomly divided into five groups (*n* = 8 per group), with each mouse as the experimental unit. Fecal suspensions were orally gavaged into recipient mice (0.2 mL of 100 mg/mL homogenized fecal slurry every other day). Young mice received saline (Young). Aged mice were randomly assigned to four groups, balanced by body weight: saline control (Aged), FMT from untreated young donors (yFMT), FMT from BL-intervened donors (BLyFMT), or combined BL diet and FMT from BL-intervened donors (BLyFMT + BL). All treatments lasted 70 days, with final assessments performed on day 130. During the experimental period, two mice from each aged group were excluded from the final analysis due to mortality. To maintain equal group sizes, two mice from the Young group were also randomly excluded. The final sample size was *n* = 6 per group for all statistical analyses.

At the endpoint of the study, mice were deeply anesthetized by CO_2_ inhalation, followed by blood collection via retro-orbital sinus puncture. Subsequently, mice were euthanized by cervical dislocation to ensure death, in accordance with the AVMA Guidelines for the Euthanasia of Animals (2020 edition).

### 2.2. qPCR for Bacterial Abundance and Gene Expression

Fecal genomic DNA from both human volunteers and experimental mice was extracted using the TIANamp Stool DNA Kit (TIANGEN Biotech, Beijing, China) following the manufacturer’s protocol with minor modifications. Absolute quantification was performed using specific primers. For tissue gene expression analysis, total RNA was extracted from the hypothalamus and testis using TRIzol reagent and reverse-transcribed into cDNA using the PrimeScript RT Master Mix (TaKaRa). SYBR Green qPCR was performed on the quantitative PCR instrument (Roche 480, Roche, Basel, Switzerland). Relative mRNA expression was calculated using the 2^ΔΔCt^ method [29]. All primer sequences used in this study, including those for bacterial 16S rRNA genes and mouse target genes, are listed in Supplementary Table S2.

### 2.3. Enzymatic and Oxidative Stress Assays

Activities of superoxide dismutase (SOD), glutathione peroxidase (GPx), glutathione (GSH), glutathione disulfide (GSSG), and the level of lipid peroxidation product malondialdehyde (MDA) were measured using commercial kits (Jiancheng Nanjing, Nanjing, China). Activities of β-galactosidase, monoamine oxidase B (MAO-B), serum reproductive hormones (LH, FSH, testosterone), cortical serotonin, corticosterone, and inflammatory markers (IL-6, IL-1β, and TNF-α) were quantified by ELISA kits (MEIMIAN, Yancheng, China).

### 2.4. Non-Targeted Metabolic Profiling

Hippocampal tissues were rapidly dissected, snap-frozen in liquid nitrogen, and stored at −80 °C until analysis. Hippocampal tissue was homogenized in 800 μL of pre-chilled methanol-water mixtures (4:1, *v*/*v*) containing 0.1% formic acid, followed by vortexing (30 s) and centrifugation (14,000× *g*, 15 min, 4 °C). The supernatant was collected and filtered through a 0.22-μm membrane prior to UPLC-MS/MS analysis. Chromatographic separation was performed on an ACQUITY UPLC HSS T3 column (2.1 mm × 100 mm × 1.8 μm) with a gradient elution of 0.1% formic acid in water (A) and acetonitrile (B). Mass spectrometry was conducted on a Q Exactive HF-X instrument (Thermo Fisher Scientific, Waltham, MA, USA) in both positive and negative electrospray ionization modes. Nontargeted metabolomic profiling was performed by using ultra-high-performance liquid chromatography–tandem mass spectrometry platforms. Peaks corresponding to individual metabolites were quantified using the area under the curve. Raw area counts for each metabolite were normalized to correct for variation resulting from interday instrument tuning differences. This normalization preserved biological variation between samples while enabling comparison of metabolites with widely different raw peak intensities on a comparable scale. Missing values were imputed with the observed minimum value after normalization. Identified metabolites were annotated based on retention index, accurate mass, and fragmentation patterns [3].

### 2.5. Behavioral Assessments

Behavioral assessments were performed by experimenters blinded to the treatment groups. Behavioral tests were conducted in a room with dim lighting during the light phase. Mice were habituated to the testing room for at least 30 min before each test.

#### 2.5.1. Spontaneous Alternation in the Y-Maze

To assess short-term working memory and hippocampal-dependent spatial exploration, spontaneous alternation behavior was assessed in the Y-maze test as previously described. Mice were individually placed at the end of one arm and allowed to explore all three arms for 5 min freely. An alternation was defined as a sequence of consecutive entries into all three arms without repetition. Total arm entries were recorded as a measure of general locomotor activity [30].

#### 2.5.2. Novel Object Recognition

To assess short-term recognition memory, mice were subjected to the NOR task with minor modifications. Animals were habituated to the testing room for 60 min before testing. The test was conducted in an open arena. On the day before the trial, the mice underwent two 10-min habituation sessions in the empty arena, three hours apart. On the testing day, the mice were first exposed to two identical objects placed in opposite corners of the arena for 10 min. Three hours later, one of the familiar objects was replaced with a novel object. The mice were then allowed to explore the arena for a further 10 min [31].

#### 2.5.3. Passive Avoidance Test

The experiment was conducted using a two-chamber apparatus comprising light and dark compartments. On the day of the experiment, each animal was placed in the illuminated compartment, oriented in a direction that was opposite to the door. Each mouse was placed in the illuminated compartment facing away from the door. Following a period of 30 s, the door opened and, upon the mouse entering the dark compartment with all four paws, the door closed automatically, and a foot shock was delivered. After this, the retention process was subjected to a 24 h period of testing under conditions that were identical to the initial setup, with the exception that no shock was administered during this phase. The latency to enter the dark compartment was recorded for a period of up to 5 min [32].

#### 2.5.4. Light–Dark Box

To assess anxiety-like behavior and approach-avoidance conflict, the light–dark box test was performed. The apparatus consisted of two connected compartments: a brightly illuminated chamber and a dark chamber. Mice were individually placed in the center of the light compartment facing away from the dark compartment and allowed to freely explore both compartments for 10 min. The time spent in the light compartment, the number of transitions between compartments, and the latency to first enter the dark compartment were recorded. The apparatus was cleaned with 70% ethanol between trials [33].

#### 2.5.5. Elevated Plus Maze

To assess anxiety-like behavior, the elevated plus maze test was conducted as previously described. The EPM apparatus was elevated 1 m above the floor and consisted of a gray cross-shaped maze with two open arms and two enclosed arms, connected by a central square. Mice were individually placed in the center square facing an open arm and allowed to explore freely for 5 min. Testing was performed under red light to minimize visual stress. The time spent in open arms, the number of open arm entries, and the total arm entries were recorded. The percentage of time spent in open arms and the percentage of open arm entries were calculated as indicators of anxiety-like behavior. The apparatus was thoroughly cleaned with 70% ethanol between animals [33].

### 2.6. Hematoxylin and Eosin (H&E) Staining

The testes of the mice were extracted rapidly under deep anaesthesia and preserved in testicular tissue fixation buffer for a 24 h period. Tissues were subsequently dehydrated via an ethanol series, and the fixed testes were embedded in paraffin and then sectioned. Paraffin sections (5-μm-thick) were then subjected to H&E staining. The histological analysis was conducted using a digital panoramic scanner [34].

### 2.7. Semen Evaluation

The cauda epididymis sperm from mice was collected and prepared in accordance with the previously established protocol. Briefly, two caudal epididymis samples were placed into TYH medium, then the cauda epididymis was divided into three pieces and incubated in a 5% CO_2_ incubator at 37 °C for 5 min. A volume of 10 μL of sperm suspension was used for evaluating sperm count and motility. This evaluation was conducted using a computer-assisted semen analysis (CASA). The evaluation focused on sperm concentration, viability, progressive motility, total motility, curvilinear velocity (VCL), and abnormality rate [12].

### 2.8. Fertility Assessments

To evaluate reproductive competence, aged male mice from each group (*n* = 6) were individually co-housed with a sexually mature, proven-fertile young female (8–10 weeks old) for up to 14 days. The presence of a vaginal plug was checked daily as an indicator of successful mating. Pregnant females were monitored until parturition, and the following parameters were recorded, including conception latency (days from co-housing to plug detection), litter size, and pup birth weight.

### 2.9. Statistical Analysis

Data analysis and visualization were performed with GraphPad Prism 10.4 for Windows. Before statistical analysis, normality and homogeneity of variance were verified using Shapiro–Wilk and Levene’s tests, respectively. The one-way ANOVA followed by Tukey’s post hoc test was applied to multiple treatment comparisons. Data are presented as mean ± SEM. Different lowercase letters above bars denote statistically significant differences among groups.

## 3. Results

### 3.1. The yFMT Treatments Mitigate Age-Associated Systemic Oxidative Stress Damage and Inflammation

**Figure 1:**
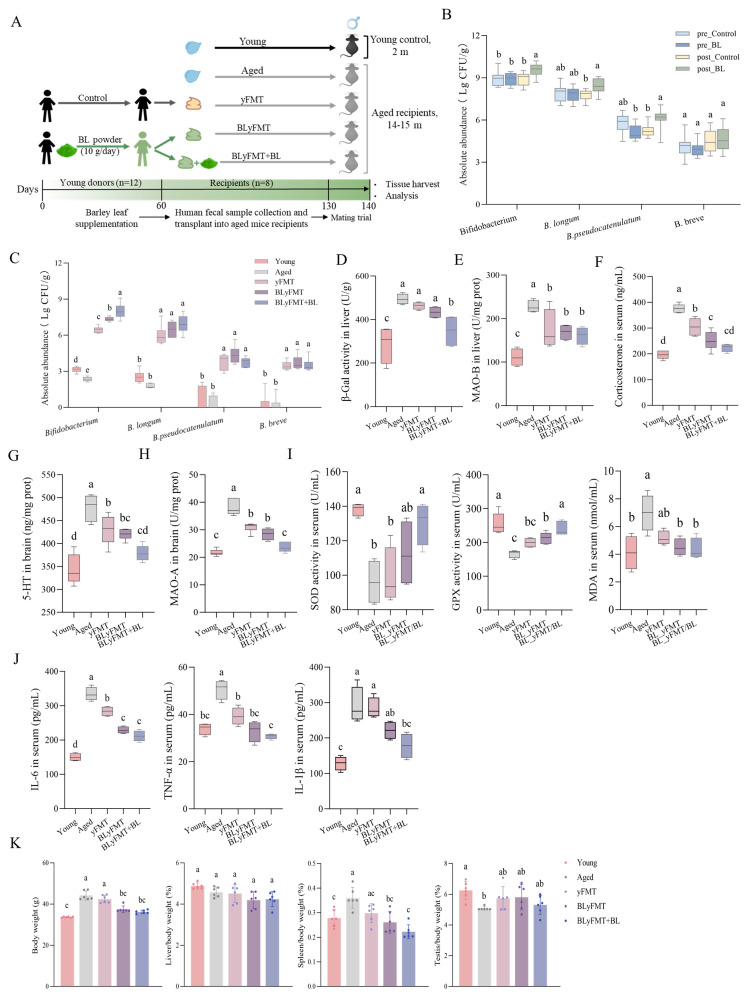
Young fecal microbiota transplantation ameliorates systemic aging markers in aged mice. (**A**) Schematic diagram of the experimental design. Stool samples were collected from healthy young volunteers either without (control) or following a 60–day BL dietary intervention. Fecal suspensions were orally gavaged into recipient mice. Young mice (2 months old) received saline (Young). Aged mice (14–15 months old at baseline) were randomly assigned to four groups: saline control (Aged), yFMT, BLyFMT, or BLyFMT + BL. All treatments lasted 70 days, with final assessments performed on day 130. (**B**) Absolute abundances of *Bifidobacterium* spp., *B. longum*, *B. pseudocatenulatum*, and *B. breve* in human donor feces. (**C**) Absolute abundances of *Bifidobacterium* spp., *B. longum*, *B. pseudocatenulatum*, and *B. breve* in recipient mouse feces. (**D**,**E**) Hepatic senescence-associated β-galactosidase activity and MAO-B activity. (**F**) Serum corticosterone levels. (**G**,**H**) Cortical serotonin (5-HT) concentration and MAO-A activity in brain. (**I**) Serum oxidative stress markers: SOD and GPx activities, and MDA content. (**J**) Serum pro-inflammatory cytokines (IL-6, IL-1β, TNF-α). (**K**) Effects of interventions on body weight, relative liver weight, relative spleen weight, and relative gonad weight. Data are presented as mean ± SEM. *n* = 6 independent mice per group. Different lowercase letters above bars denote statistically significant differences among groups (*p* < 0.05, one-way ANOVA with post hoc Tukey test).

As shown in the experimental design (Figure 1A), we aimed to determine whether the fertility decline associated with aging could be reversed through various young fecal microbiota transplantation treatments, and whether a combined treatment would have the most pronounced effect. During the process of ageing, the bacterial genus *Bifidobacterium* has been observed to be present at a lower abundance in comparison to younger individuals [35,36]. This decline has been demonstrated to be strongly correlated with the presence of inflammation, intestinal barrier dysfunction, and systemic metabolic dysregulation. A study of the fecal microbiome of semi-supercentenarians found a shift towards the health-associated bacterial genus *Bifidobacterium* [37]. Thus, the donor fecal samples were selected based on a high relative abundance of *Bifidobacterium* with prior BL dietary intervention. Firstly, we collected fecal samples from young, healthy volunteers who had received BL dietary intervention or not. These fecal samples were used to perform the yFMT treatments in aged mice. Following BL dietary intervention, we observed a significant increase in the absolute abundance of *Bifidobacterium* in both human donor feces and recipient mouse stools (Figure 1B,C). To evaluate the systemic anti-aging effects of our interventions, we first assessed markers of cellular senescence, oxidative stress, and inflammation in aged mice. Aged mice exhibited significantly elevated hepatic activity of senescence-associated β-galactosidase and MAO-B compared to young controls (Figure 1D,E). Notably, when compared to the Young group, the corticosterone level in serum and 5-HT content in brain from aged mice were both significantly enhanced, followed by dramatically increased activity of its catabolic enzymes MAO-A (Figure 1F–H). Additionally, age triggered production of the oxidative stress markers and pro-inflammatory cytokines in serum, which were partially reduced by yFMT treatment but showed a more pronounced and significant decrease in BLyFMT or the combined regimen of BLyFMT + BL (Figure 1I,J). Interestingly, this enhanced efficacy was accompanied by a significant increase in the absolute abundance of *Bifidobacterium* in both human donor feces and recipient mouse stools following barley leaf dietary intervention.

### 3.2. Hippocampal Metabolome Is Reprogrammed by Combined yFMT Interventions

Given the central role of the hippocampus in cognitive and emotional functions, we performed non-targeted metabolomics to investigate the neural metabolic landscape [38].

**Figure 2:**
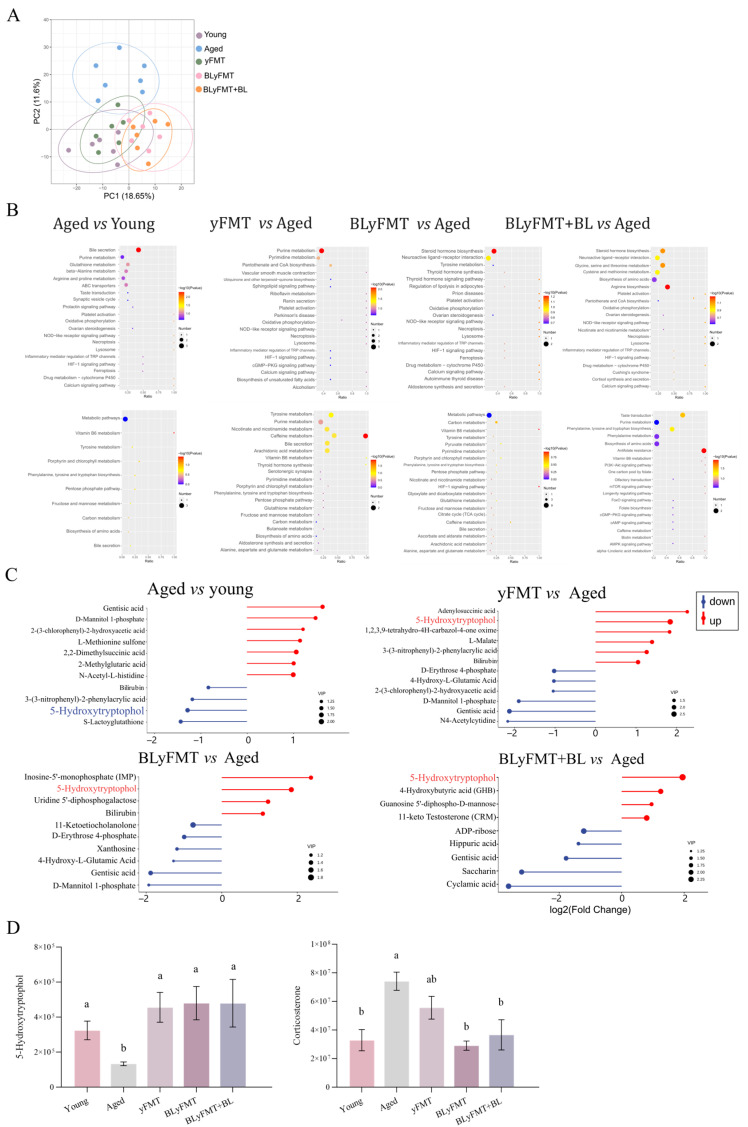
Untargeted metabolomics assessment of the hippocampus by BLyFMT and dietary barley leaf supplementation. (**A**) Principal component analysis showing the effect of FMT treatments on hippocampal metabolome based on the Bray–Curtis distance matrix. (**B**) KEGG pathway enrichment analysis of differentially abundant metabolites. (**C**) Heatmap showing expression patterns of significantly altered metabolites across groups. (**D**) Hippocampal levels of 5-hydroxytryptophol and corticosterone. al microbiota transplantation ameliorates systemic aging markers in aged mice. *n* = 6 independent mice per group. Different lowercase letters above bars denote statistically significant differences among groups (*p* < 0.05, one-way ANOVA with post hoc Tukey test).

Principal component analysis of the hippocampal metabolome showed a clear separation between the Young and Aged groups along PC1 (18.65% variance) and PC2 (11.6% variance). Notably, all three intervention groups (yFMT, BLyFMT, and BLyFMT + BL) exhibited a progressive shift toward the Young cluster, with the BLyFMT + BL group clustering the closest proximity to the metabolic profile in young individuals (Figure 2A). Multivariate analysis revealed a clear separation between the aged group and all intervention groups (Figure S1). KEGG pathway enrichment analysis showed that aging profoundly disrupted metabolic pathways, which were progressively reversed by interventions. While aged mice showed significant downregulation in pathways like tyrosine and purine metabolism, the BLyFMT treatment and combinatorial BLyFMT + BL treatment induced the most robust reprogramming, with steroid hormone biosynthesis and neuroactive ligand–receptor interaction emerging as enriched pathways, followed by significant restoration of arginine biosynthesis in the BLyFMT + BL group, indicating a comprehensive metabolic rejuvenation of the hippocampus (Figure 2B). Moreover, differentially expressed metabolite analysis showed that the hippocampal 5-hydroxytryptophol, a serotonin derived metabolite implicated in the fine-tuning of serotonergic signaling [39], was markedly reduced in aged mice relative to young controls. All interventions progressively restored 5-hydroxytryptophol level, with the BLyFMT + BL group exhibiting the most pronounced elevation, surpassing even the young baseline. Given that 5-hydroxytryptophol can modulate neuronal excitability and promote antioxidant responses at physiological concentrations, its restoration aligns with the observed improvements in cortical serotonin homeostasis, reduced hippocampal MAO-B activity, and ameliorated cognitive-affective behaviors (Figure 2C) [40]. Notably, the rise in hippocampal 5-hydroxytryptophol across intervention groups was inversely correlated with corticosterone levels, suggesting a coordinated rebalancing of serotonergic tone and HPG axis activity (Figure 2D).

### 3.3. Aging-Associated Behavioral Deficits in Aged Mice Are Rescued by BLyFMT with Dietary Barley Leaf

**Figure 3:**
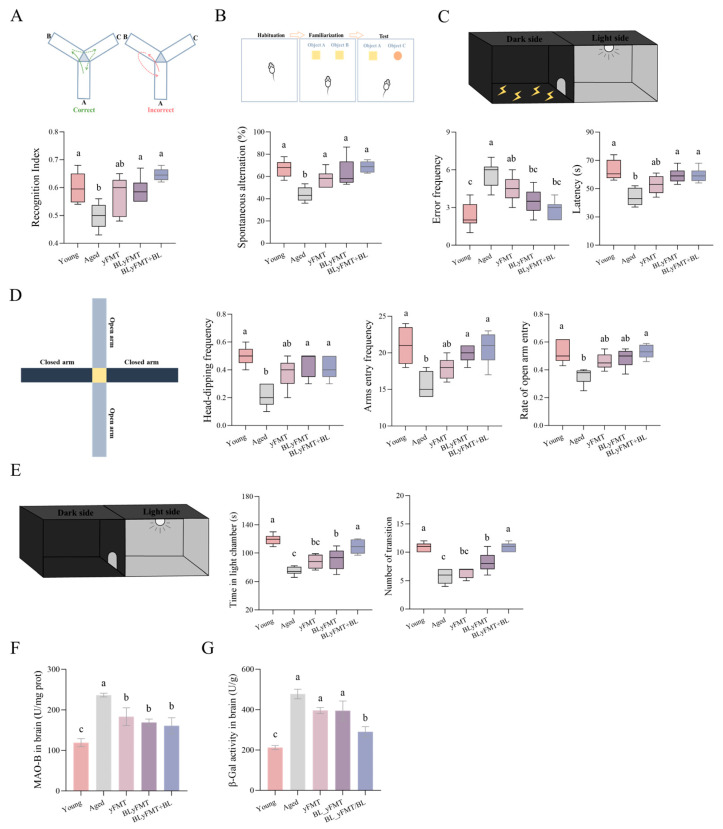
Barley leaf-augmented yFMT improves behavioral declines and reduces neuroinflammation in aged mice. Behavioral assessments: (**A**) Y-maze spontaneous alternation; (**B**) Novel object recognition; (**C**) Passive avoidance latency; (**D**) Elevated plus maze; (**E**) Light–dark box test. (**F**,**G**) Hippocampal MAO-B activity and β-galactosidase activity. *n* = 6 independent mice per group. Different lowercase letters above bars denote statistically significant differences among groups (*p* < 0.05, one-way ANOVA with post hoc Tukey test).

We next examined whether the observed biochemical and metabolic improvements translated into functional benefits in behavior. Aged mice displayed significant cognitive impairments, as shown by reduced spontaneous alternation in the Y-maze (Figure 3A) and a lower recognition index in the novel object recognition test (Figure 3B). Their fear memory was also compromised, evidenced by a shortened latency in the passive avoidance test (Figure 3C). All these cognitive deficits were ameliorated by the interventions, with the BLyFMT + BL group showing the most robust and consistent rescue effect, performing comparably to young mice. Furthermore, aged mice exhibited heightened anxiety-like behaviors, spending less time in the open arms of the elevated plus maze (Figure 3D) and in the light compartment of the light–dark box (Figure 3E). These anxiety phenotypes were significantly alleviated by BLyFMT + BL. This behavioral recovery was associated with a marked reduction in hippocampal senescence β-galactosidase activity and MAO-B activity (Figure 3F,G). These data collectively demonstrate that barley leaf supplementation synergistically enhances the efficacy of yFMT, driving a restoration of cognitive function through coordinated mitigation of neural senescence, oxidative stress, and inflammation.

### 3.4. Testicular Structure and Spermatogenic Function Are Restored by Combinatorial Intervention

**Figure 4:**
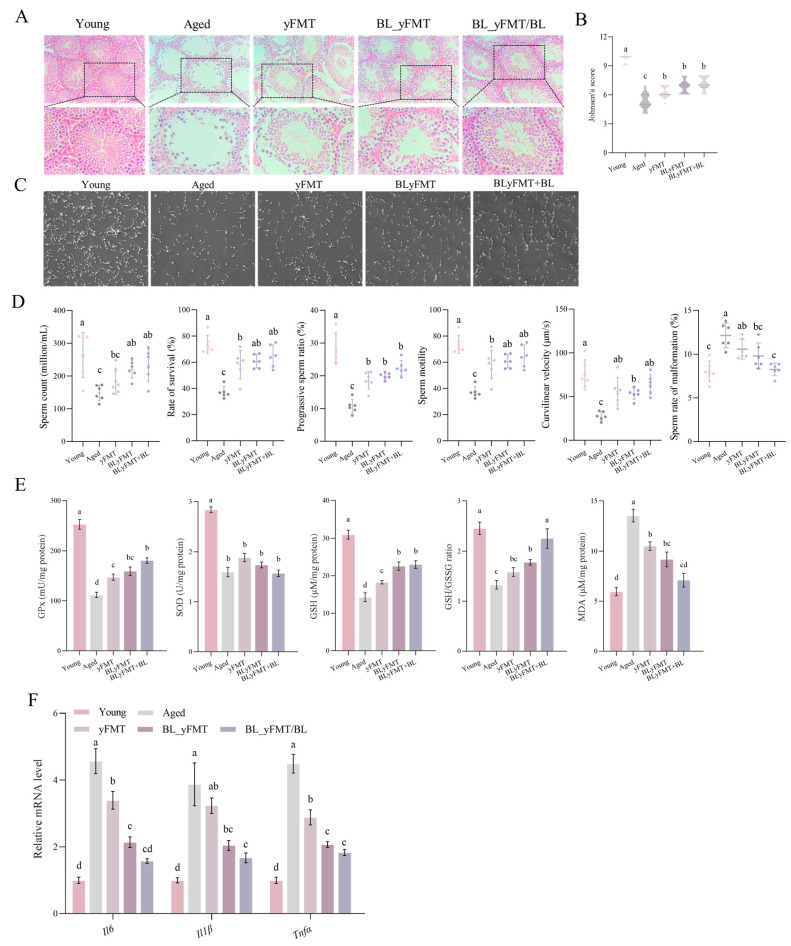
Restoration of testicular structure and function by BLyFMT combined with barley leaf intervention. (**A**) Representative H&E-stained testicular sections. (**B**) Johnsen’s scoring of seminiferous tubule integrity. (**C**) Sperm morphology and motility assessment. (**D**) Sperm parameters: density, viability, progressive motility, total motility, curvilinear velocity, and abnormality rate. (**E**) Testicular antioxidant status: GPx and SOD activities, GSH content, GSH/GSSG ratio, and MDA levels. (**F**) mRNA expression of inflammatory genes (*Il6*, *Il1β*, *Tnfα*) in testis tissue. *n* = 6 independent mice per group. Different lowercase letters above bars denote statistically significant differences among groups (*p* < 0.05, one-way ANOVA with post hoc Tukey test).

Given that the combinatorial intervention not only reversed age-associated hippocampal metabolic dysregulation and behavioral decline but also normalized systemic stress and inflammation, we hypothesized that these benefits would extend to the male reproductive system, a compartment highly sensitive to neuroendocrine and oxidative perturbations during aging [41]. Consistent with this, aged mice exhibited profound testicular degeneration, including disrupted seminiferous tubule architecture with germ cell depletion and vacuolization (Figure 4A), reflected in a significantly reduced Johnsen’s score (Figure 4B), as well as impaired spermatogenic output, manifesting as decreased sperm density, viability, progressive motility, and curvilinear velocity alongside elevated morphological abnormalities (Figure 4C,D). While the yFMT partially mitigated these deficits, which were evidenced by modest improvements in tubular integrity, sperm density, and viability. The addition of barley leaf to the donor diet (BLyFMT) significantly prevented this damage, with higher Johnsen scores and enhanced progressive motility compared to yFMT, while residual impairments persisted in sperm velocity and abnormality rates. Strikingly, the BLyFMT + BL treatment produced the most comprehensive restoration as seminiferous epithelium architecture closely approximated that of young mice (Figure 4A), Johnsen’s score approached youthful levels (Figure 4B), and all measured sperm parameters were significantly superior to both yFMT and BLyFMT groups (Figure 4C,D), collectively indicating near-complete functional rejuvenation. This improvement was paralleled by a graded amelioration of testicular redox imbalance, as SOD and GPx activities, GSH content, and the GSH/GSSG ratio increased progressively, while MDA levels declined correspondingly (Figure 4E). Likewise, higher mRNA expression of *Il6*, *Il1β*, and *Tnfα* in aged mice was suppressed with the lowest levels observed in the BLyFMT + BL group (Figure 4F). Thus, the synergistic effect of barley leaf enhanced FMT and dietary supplementation jointly drives a rescue of testicular structure and function, which supported that the intestinal–microbiome–neuroendocrine axis as a unified target for multi-systemic aging intervention.

### 3.5. Reproductive Competence and HPG Axis of Aged Male Mice Is Sensitive to the yFMT Modulators

**Figure 5:**
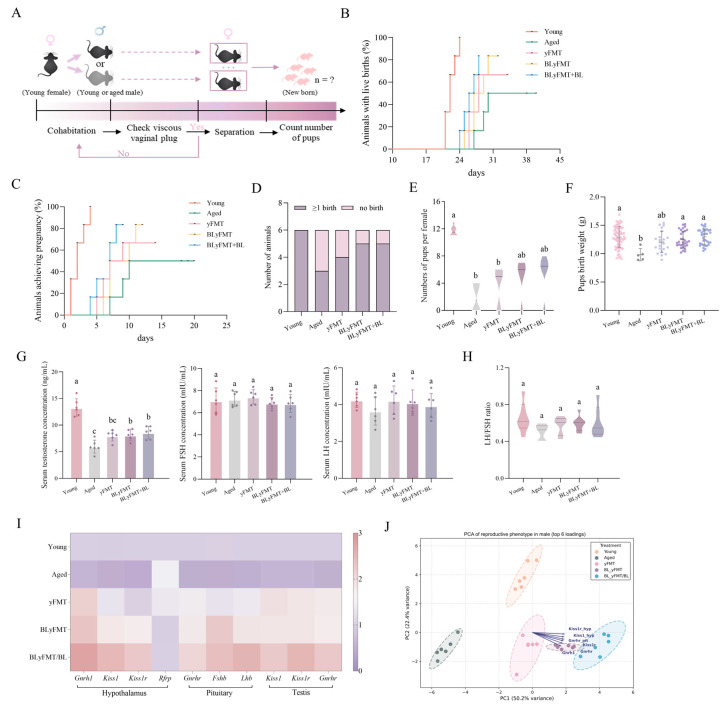
Enhanced reproductive competence in aged male mice following BLyFMT and barley leaf co-intervention. (**A**) Experimental design for assessing the fertility of aged male mice. Each experimental male was co-housed with a single young, fertile female. Pregnancy and live birth outcomes were recorded per female. (**B**) Proportion of young female mice achieving pregnancy after mating with aged males. (**C**) Proportion of young female mice achieving live birth. (**D**) Cumulative probability of live birth over time. (**E**) Total number of pups born per pregnant young female mated with aged males. (**F**) Birth weight of pups born to young females mated with aged males. Data are presented as mean ± SEM; no significant differences were observed between groups (*p* > 0.05). (**G**) Serum testosterone, LH, and FSH levels. (**H**) LH/FSH ratio. (**I**) Heatmap of mRNA expression of HPG axis-related genes. (**J**) PCA integrating reproductive performance and hormonal profiles. *n* = 6 independent mice per group. Different lowercase letters above bars denote statistically significant differences among groups (*p* < 0.05, one-way ANOVA with post hoc Tukey test).

To ascertain whether the enhancements observed in testicular structure and function were indicative of genuine reproductive capacity, a controlled breeding trial was conducted [42]. In this trial, aged male mice were paired with young female mice (Figure 5A). Aged males exhibited severely impaired fertility, characterized by delayed conception, reduced total number of pups per female, and lower average pup birth weight (Figure 5B–F). The yFMT treatment accelerated the onset of pregnancy and increased litter size compared to the aged control. The BLyFMT treatment further improved outcomes, yielding shorter conception latency and higher pup numbers than in the yFMT group (*p* < 0.05), though pup weight and consistency of delivery still lagged. After receiving BLyFMT + BL intervention, the total pup number was significantly increased compared to that of BLyFMT, and pup birth weight approached that of the young control. Additionally, hormonal levels, including luteinizing hormone (LH), follicle stimulating hormone (FSH), and testosterone, were detected. Notably, the level of testosterone inhibited in aging was progressively normalized, and a higher LH/FSH ratio was observed in the BLyFMT + BL group compared to both yFMT and BLyFMT (Figure 5G,H). At the transcriptional level, mRNA expression of HPG axis genes [43,44,45], including hypothalamic *Gnrh1*, *Kiss1*, *Kiss1r*, and *Rfrp*, pituitary *Gnrhr*, *Lhb* and *Fshb*, and testicular *Kiss1*, *Kiss1r*, and *Gnrhr*, were dysregulated in aged mice but restored, with BLyFMT + BL eliciting the most coherent upregulation across all three compartments (Figure 5I). Principal component analysis (PCA) of the integrated reproductive phenotype showed a clear experiment-related batch effect along the PC1 (50.2% variance), distinctly separated from aged groups, underscoring that dietary barley leaf synergistically amplifies the rejuvenating capacity of young FMT to restore holistic male reproductive competence (Figure 5J).

## 4. Discussion

In this study, we examined the contribution of the host microbiota from health young human donors with or without BL-dietary intervention to age-related physiologies of cognitive decline and fertility impairment by using naturally aged male mice. The modulation of counts of genus *Bifidobacterium* and several bifidobacterial species is a primary criterion for selecting dietary interventions targeting the gut microbiota because it is a core colonizing genus in the host and greatly influenced by age [46]. Interestingly, dietary BL supplementation markedly elevated the absolute abundances of *Bifidobacterium* spp., *B. longum*, *B. pseudocatenulatum*, and *B. breve* in human donor fecal samples. Subsequent transplantation into recipient mice confirmed the effective engraftment of these BL-enriched taxa, which validated the potential of this pre-conditioning strategy. In those recipient mice, we further found that these yFMT treatments ameliorated age-related symptoms such as the accumulation of inflammatory factors and oxidative stress injury to different extents. Additionally, the restored hippocampal metabolome coincided with the improvements in cognitive deficits under different yFMT treatments. Moreover, we uncovered the protective effects of yFMT with the augmentation of *Bifidobacterium* on testicular antioxidant function, semen quality, and the fertility decline of aging males. Our findings demonstrated that a combinatorial strategy, involving the priming of young human donor microbiota with BL and the supplementation with BL, synergistically enhances the efficacy of yFMT to achieve a holistic rejuvenation of both neural and reproductive function in aged male mice. This dual neuro-reproductive protection is achieved through a systemic reduction in oxidative stress and inflammation. Furthermore, it has been shown to preserve testicular structure, support healthy sperm production, and ultimately enhance male fertility [47].

The central finding of our work is the profound synergy between donor and recipient interventions. While standard yFMT provided modest benefits, and BLyFMT treatment showed further improvement, only the BLyFMT + BL treatment achieved near-complete functional restoration across all measured parameters. The data strongly suggest that BL pre-conditioning of the donor shapes a more resilient and therapeutically potent microbial consortium, while concurrent dietary BL in the recipient creates a favorable metabolic niche that promotes its engraftment and functional output. This aligns with the concept of colonization resistance, where the aged gut environment typically rejects microbes due to inflammation [16]. Our strategy overcomes this by providing a niche rich in fermentable substrates and high level of *Bifidobacterium* microecosystem that supports the transplanted taxa.

The current study described a microbiome-based rejuvenation derived from interaction of dietary component by human gut communities. Parker et al. demonstrated that young-to-old FMT could reverse hallmarks of aging in the gut, eye, and brain [6], and Boehme et al. discovered similar benefits for restoring aging-associated immune and neurocognitive decline, these studies conducted solely microbial transfer [48]. To address the fundamental challenge of poor engraftment in aged hosts, our work underscored the necessity of combining microbial transplantation with targeted dietary modulation. We demonstrated that dietary pre-conditioning of both donor and recipient could dramatically enhance FMT efficacy, achieving more comprehensive functional restoration. Furthermore, we provided the evidence of synchronized rejuvenation of both the nervous and reproductive systems through a gut–microbiome–neuroendocrine axis. This microbe–host interaction approach is particularly important, as clinical observations have shown that cognitive decline and reproductive dysfunction often simultaneous in elderly men [49,50,51].

Our results provide a compelling mechanistic link between the gut microbiome and the neuro-reproductive axis, a concept consistent with human observational studies linking gut dysbiosis to reproductive hormone disturbances [41]. Increasingly, evidence has established a clear longitudinal association between declining testosterone and accelerated cognitive decline in men [49,50,51,52]. Our results presented herein suggest a potential causal pathway for this clinical observation, namely that an aged, pro-inflammatory gut microbiome exerts a deleterious effect on the brain and testicles, causing oxidative stress at the tissue level. This, in turn, results in impaired neuronal function and disruption to HPG-axis signalling. The concurrent alleviation of these parallel pathologies accompanies the restoration of the gastrointestinal ecosystem. Specifically, *Bifidobacterium*-derived metabolites such as SCFAs and tryptophan derivatives might directly modulate HPG axis signaling and protect against neuroinflammation through multiple mechanisms [53].

The inverse correlation observed between 5-hydroxytryptophol, a serotonin metabolite with emerging roles in neuronal redox regulation, and serum corticosterone further highlights the intricate crosstalk between the gut, brain, and gonad. The restoration of 5-hydroxytryptophol to levels exceeding those of young controls is a particularly intriguing finding. Its physiological elevation in the context of improved redox status may represent an adaptive, protective response that modulates serotonergic signaling and contributes to the observed behavioral improvements. This challenges the simplistic view of serotonin catabolites as purely detrimental and suggests their role is highly context-dependent.

Our diet-enhanced FMT strategy partially addresses these challenges of current FMT protocols for age-related conditions through utilizing a natural, widely available dietary component that can be easily incorporated into daily nutrition and providing a dual benefit of microbiota modulation. Moreover, our identification of *Bifidobacterium* enrichment as a key biomarker of treatment response provides a measurable target for optimizing personalized interventions.

While our findings are robust, several limitations warrant consideration. First, the study was conducted exclusively in male mice. Future work must explore whether this strategy is equally effective in females. Second, although we used human donor samples for FMT, the recipients were mice, and the translational leap to humans requires careful validation in clinical trials. Third, while we identified *Bifidobacterium* enrichment as a key feature, the precise microbial strains and their derived metabolites responsible for the observed effects remain to be fully elucidated. Future studies should employ shotgun metagenomics and targeted metabolomics to pinpoint specific strains and metabolites. Moreover, the impact of sex-specific factors that affect the response to reproductive protection and neuroprotection required to be uncovered. Above all, in this study, we present a diet-enhanced yFMT approach that synchronizes the rejuvenation of neural and reproductive systems in aging male mice. We demonstrate that a natural dietary component can significantly enhance the effectiveness of young microbiota transplantation, particularly by boosting the levels of *Bifidobacterium*. This work provides a mechanistic basis for microbiome-targeted combination therapies to combat multi-system aging.

## 5. Conclusions

In summary, this study demonstrates that barley leaf-augmented young fecal microbiota transplantation synergistically ameliorates cognitive decline and reproductive senescence in aged male mice. The combined strategy of donor preconditioning and recipient barley leaf supplementation significantly enhances Bifidobacterium engraftment, reprograms hippocampal metabolomic profiles, restores testicular structure and spermatogenesis, and rejuvenates HPG axis activity. These benefits are mediated by the coordinated reduction of systemic oxidative stress and inflammation, alongside the restoration of key metabolites such as 5-hydroxytryptophol. Our findings establish Bifidobacterium enrichment as a key efficacy biomarker and provide a mechanistic basis for diet-enhanced FMT as a promising translational strategy to address concurrent neural and reproductive decline in aging populations.

## Data Availability

The original contributions presented in this study are included in the article. Further inquiries can be directed to the corresponding author.

## Supplementary Materials

The following supporting information can be downloaded at: https://www.mdpi.com/article/10.3390/nu18081193/s1.

## Abbreviations

The following abbreviations are used in this manuscript:

BL	Barley leaves
FMT	Fecal microbiota transplantation
yFMTs	Young fecal microbiota transplants
BLyFMT	FMT from BL-intervened donors
BLyFMT + BL	Combined BL diet + FMT from BL-intervened donors
HPG	Hypothalamic–pituitary–gonadal
*B. longum*	*Bifidobacterium longum*
SOD	Superoxide dismutase
GPx	Glutathione peroxidase
GSH	Glutathione
GSSG	Glutathione disulfide
MDA	Malondialdehyde
H&E	Hematoxylin and eosin
CASA	Computer-assisted semen analysis
VCL	Curvilinear velocity
MAO-B	Monoamine oxidase B
LH	Luteinizing hormone
FSH	Follicle stimulating hormone
PCA	Principal component analysis
