# Supplementary material for: Young Human-Derived Microbiota Ameliorates Cognitive Decline and Reproductive Senescence in Aged Mice
Source: Nutrients. 2026 Apr 10;18(8):1193. doi: 10.3390/nu18081193 (PMC13118330; doi:10.3390/nu18081193)
Supplement: Supplementary file 1 [file nutrients-18-01193-s001.zip › nutrients-4233034-supplementary.pdf]

Young Human-derived Microbiota Ameliorates Cognitive Decline and Reproductive Senescence in Aged Mice

Xiaoying Zhang [1], Fang Chen[1], Yinghua Luo[1], Daotong Li[1], Junfu Ji[1], Lingjun Ma[1], Chen Ma[1*] and Xiaosong Hu[1*]

[1] College of Food Science and Nutritional Engineering, National Engineering Research Center for Fruit and Vegetable Processing, Key Laboratory of Fruit and Vegetables Processing Ministry of Agriculture, Engineering Research Centre for Engineering Vegetables Processing, Ministry of Education, China Agricultural University, Beijing 100083, China

* Correspondence: machen21@cau.edu.cn; huxiaos@263.net

This file includes:

Figure S1. Untargeted metabolomics assessment of the hippocampus by BLyFMT and dietary barley leaf supplementation. Multivariate analysis (Partial Least Squares Discriminant Analysis) score plot of non-targeted hippocampal metabolomics (n = 6 per group).

Table S1. Nutritional composition of barley leaf (BL) powder (per 100 g dry weight).

Table S2. Primer sequences.

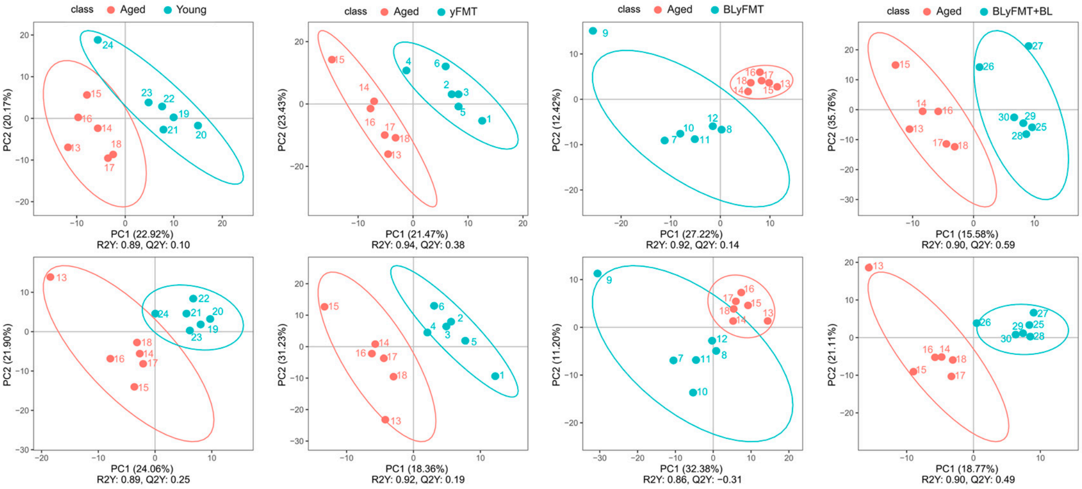

Figure S1. Untargeted metabolomics assessment of the hippocampus by BLyFMT and dietary barley leaf supplementation. Multivariate analysis (Partial Least Squares Discriminant Analysis) score plot of non-targeted hippocampal metabolomics (n = 6 per group).

Table S1. Nutritional composition of barley leaf (BL) powder (per 100 g dry weight)

Component	Content
Protein	23.9 g
Fat	2.98 g
Carbohydrate	62.3
Insoluble dietary fiber	53.6 g
Soluble dietary fiber	< 0.01
Moisture	3.99
Ash	6.8
Chlorophyll	0.30 g
Flavonoids	0.51 g

Table S2. Primer sequences

Primer name	Primer sequence
Bifidobacterium	F: 5'-GGGTGGTAATGCCGGATG-3'
	R: 5'-CCACCGTTACACCGGGAA-3'
B. longum	F: 5'-TTCCAGTTGATCGCATGGTC-3'
	R: 5'-GGGAAGCCGTATCTCTACGA-3'
B. pseudocatenulatum	F: 5'-CGGATGCTCCGACTCCT-3'
	R: 5'-CGAAGGCTTGCTCCCGAT-3'
B. breve	F: 5'-CCGGATGCTCCATCACAC-3'
	R: 5'-ACAAAGTGCCTTGCTCCCT-3'
Gnrh1	F: 5'-TCTGAGCTGCTGCTGCTCTT-3'
	R: 5'-GGTGGTGGTGGTAGTGTTGG-3'
Kiss1	F: 5'-AGTGCCTTCTGCTGCTCTTC-3'
	R: 5'-TGCTGGTGGTGTAGTAGCAG-3'
Kiss1r	F: 5'-GCTGCTGCTCTTCTGCTCTC-3'
	R: 5'-CAGGTGGTGGTGGTAGTGTT-3'
Rfrp	F: 5'-GGAGCTGCTGCTGCTCTTCT-3'
	R: 5'- GGTGGTGGTGGTAGTGTTGG-3'
Lhb	F: 5'-AGTGCCTTCTGCTGCTCTTC-3'
	R: 5'-TGCTGGTGGTGTAGTAGCAG-3'
Fshb	F: 5'-GCTGCTGCTCTTCTGCTCTC-3'
	R: 5'-CAGGTGGTGGTGGTAGTGTT-3'
Gnrhr	F: 5'-TCTGAGCTGCTGCTGCTCTT-3'
	R: 5'-GGTGGTGGTGGTAGTGTTGG-3'
